# Integrating Genetic and Single‐Cell Genomic Data to Reveal Brain Cell‐Specific Regulation of Attention‐Deficit/Hyperactivity Disorder Risk in the Prefrontal Cortex

**DOI:** 10.1002/brb3.70664

**Published:** 2025-07-07

**Authors:** Jiawei Gui, Ziyi Xia, Keqi Wan, Xiangli Dong, Weiming Sun

**Affiliations:** ^1^ Department of Rehabilitation Medicine, The First Affiliated Hospital, Jiangxi Medical College Nanchang University Nanchang China; ^2^ Postdoctoral Research Station, The First Affiliated Hospital, Jiangxi Medical College Nanchang University Nanchang China; ^3^ HuanKui Academy, Jiangxi Medical College Nanchang University Nanchang China; ^4^ The First Clinical Medical College, Jiangxi Medical College Nanchang University Nanchang China; ^5^ Department of Psychosomatic Medicine, The Second Affiliated Hospital, Jiangxi Medical College Nanchang University Nanchang China

**Keywords:** attention‐deficit/hyperactivity disorder, Mendelian randomization, prefrontal cortex, single‐cell

## Abstract

**Introduction:**

The risk of attention‐deficit/hyperactivity disorder (ADHD) may involve genetic regulation by specific brain cells in the prefrontal cortex, but the causal associations are currently unclear.

**Methods:**

We integrated single‐cell cis‐expression quantitative trait loci (cis‐eQTLs) from the prefrontal cortex with ADHD genome‐wide association studies (GWASs). Using Mendelian randomization (MR) and Bayesian colocalization analyses, we assessed how brain cell‐specific gene expression regulation affects ADHD susceptibility. We also examined the association between brain cell‐type proportion and ADHD risk and used bioinformatics analyses to explore risk gene functions and identify potential drug‐repurposing targets.

**Results:**

Single‐cell eQTL MR analysis revealed that brain cell‐specific gene regulation was causally linked to ADHD risk. For example, astrocyte‐specific VIM expression was significantly associated with increased ADHD risk (*β* = 0.167, SE = 0.0388, *p* = 1.63 × 10^−^⁵). Further MR analysis of ADHD subtypes revealed that certain associations exhibited stronger causal effects in childhood, late‐diagnosed, or persistent ADHD. Bayesian colocalization analysis further supported 24 unique genes, including VIM in astrocytes (PPH4 = 90.8%), which showed strong evidence of shared genetic signals with ADHD. The proportions of inhibitory neurons and oligodendrocytes in the prefrontal cortex were associated with specific ADHD subtypes. Bioinformatics analyses showed that risk genes were enriched in certain brain cell types and pathways relevant to ADHD pathogenesis, aligning with MR findings.

**Conclusion:**

Our results identify 24 cell‐specific genes in the prefrontal cortex that may mediate ADHD risk and highlight promising molecular targets for therapeutic development.

## Graphical abstract

This study uncovers causal associations between specific brain cells in the prefrontal cortex and ADHD risk through single‐cell eQTL MR and Bayesian analyses, advancing our understanding of ADHD's neurobiology. Our findings highlight novel therapeutic targets, paving the way for new treatment strategies in ADHD management.

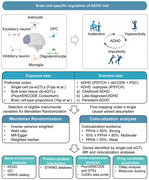

**Abbreviations**
ADHD: Attention‐deficit/hyperactivity disorder
cis‐eQTL: cis‐expression quantitative trait loci
CMap: Connectivity Map
CTP: cell‐type proportion
DLPFC: dorsolateral prefrontal cortex
FDR: false discovery rate
GABA: gamma‐aminobutyric acid
GO: Gene Ontology
GWAS: genome‐wide association study
IV: instrumental variable
IVW: inverse variance weighted
KEGG: Kyoto Encyclopedia of Genes and Genomes
LD: linkage disequilibrium
MR: Mendelian randomization
PPI: protein‐protein interaction
PV+: parvalbumin‐positive
SLOS: Smith–Lemli–Opitz syndrome
SNP: single nucleotide polymorphism

## 1 Introduction

Attention‐deficit/hyperactivity disorder (ADHD), a prevalent neurodevelopmental and psychiatric disorder affecting approximately 5% of children/adolescents and 2.5% of adults, is characterized by inattention, hyperactivity, and impulsivity (Faraone et al. 2024). These symptoms profoundly impact academic performance, physical/mental health, social functioning, and quality of life, with associations with severe outcomes like suicide and premature death (Posner et al. 2020). Although first‐line treatments benefit most ADHD patients, suboptimal symptom control and adverse side effects result in inadequate responses for a significant subset of patients, highlighting the need to investigate the complex etiology and pathophysiology of this disorder despite existing therapeutic options. Genetic factors are central to this effort, as twin studies demonstrate ∼80% heritability (Faraone and Larsson 2019), and genome‐wide association studies (GWASs) have identified multiple risk loci linked to susceptibility (Demontis et al. 2023; Rajagopal et al. 2022). Therefore, translating genetic findings into mechanisms and exploring the biological pathways from genetic variation to disease risk will be advantageous in uncovering the etiology of ADHD and improving clinical treatment.

In recent years, Mendelian randomization (MR) analysis and colocalization analysis have become important tools for studying the genetic factors and causal mechanisms of ADHD (Xie and Mao 2024; Wu et al. 2024; Ding et al. 2022). These methods can integrate GWAS and expression resources derived from brain tissue, thereby characterizing genes related to the genetic risk of ADHD and providing a basis for developing drug targets (Hammerschlag et al. 2020; Liu et al. 2023; C. Zhang et al. 2024). Recently, advancements in single‐cell genomics technology have enabled us to more accurately identify the associations between gene expression in specific brain cells and disease risk, thereby developing new therapeutic targets (Bryois et al. 2022; Fujita et al. 2024). However, no studies have examined systematic MR analysis of single‐cell genomes in specific brain regions to identify novel causal mediators of ADHD.

In this study, we utilized single‐cell cis‐expression quantitative trait loci (cis‐eQTLs) from the dorsolateral prefrontal cortex (DLPFC) for MR and Bayesian colocalization analyses to reveal the impact of genetic regulation of brain cell‐specific gene expression on susceptibility to ADHD and its subtypes. To our knowledge, this is the first research to apply single‐cell cis‐eQTL MR in ADHD, extending beyond traditional bulk tissue MR analyses to dissect cell type‐specific genetic mechanisms underlying the disorder. We focused on the DLPFC because its structural and functional disorders are closely related to the emergence of dysfunctions in attention, cognitive control, and working memory in ADHD patients (Hoogman et al. 2019). Furthermore, we explored the causal associations between brain cell‐type proportion (CTP) in the prefrontal cortex and the risk of ADHD. Meanwhile, we integrated gene enrichment analysis, protein‒protein interaction (PPI) network construction, and gene expression analysis to reveal the pathogenic mechanisms and expression patterns of these risk genes. Finally, Connectivity Map (CMap) analysis and molecular docking were used to predict potential target drugs to advance clinical drug development for ADHD.

## 2 Methods

**FIGURE 1:**
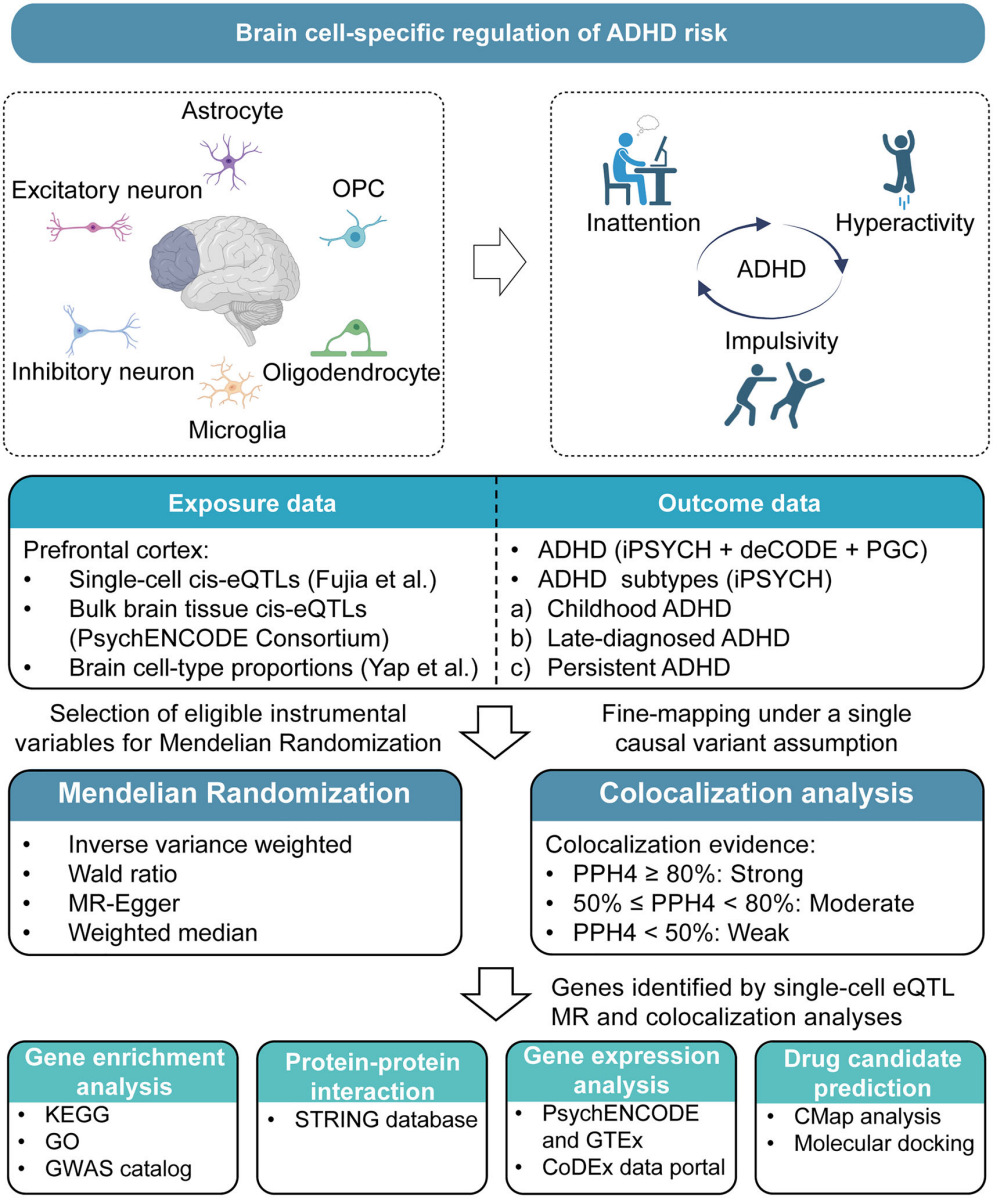
**The schematic of the study design**. ADHD, attention‐deficit/hyperactivity disorder; cis‐eQTL, cis‐expression quantitative trait loci; CMap, Connectivity Map; GO, gene ontology; GWAS, genome‐wide association study; KEGG, Kyoto Encyclopedia of Genes and Genomes; OPC, oligodendrocyte precursor cell; PPH4, posterior probability of H4. Some figure components were created using BioRender (https://biorender.com/).

A schematic of the study design is shown in **Figure** **1**, with the specific methods described below.

### 2.1 Data Sources

The single‐cell cis‐eQTL dataset was derived from a recently published study that identified cis‐eQTLs at the brain cell level using single‐nucleus RNA sequencing data from the DLPFC of 424 European ROSMAP study participants (Fujita et al. 2024). In this study, we focused on six major brain cell types (astrocytes, excitatory neurons, inhibitory neurons, microglia, oligodendrocytes, and oligodendrocyte precursor cells). The bulk tissue cis‐eQTL dataset was derived from an integrative repository conducted by the PsychENCODE Consortium (Wang et al. 2018). The cis‐eQTLs (a filter requiring genes to have an expression > 0.1 FPKM in at least 10 samples) were identified from the prefrontal cortex, involving samples from 1387 individuals. In addition, we obtained the merged PsychENCODE and GTEx brain cell gene expression matrix for the prefrontal cortex from this repository.

To focus on biologically actionable targets, we prioritized protein‐coding genes, as their expression and regulation are fundamental to the pathophysiological pathways implicated in neurodevelopmental disorders. Comprehensive gene annotation data (gencode.v45.annotation.gtf, obtained from GENCODE) were utilized to filter and select protein‐coding genes for subsequent analyses.

GWAS summary statistics for CTP in the prefrontal cortex were obtained from the study by Yap et al. (2024). They conducted a GWAS meta‐analysis (including 873 individuals of European ancestry) to identify genetic variants associated with CTP using whole‐genome sequencing or genotyping data from ROSMAP, LIBD, and UCLA_ASD. GWAS summary statistics for ADHD were collected from the largest available GWAS meta‐analysis of ADHD to date (Demontis et al. 2023). The study integrated data from the Danish iPSYCH cohort, the Icelandic deCODE cohort, and the PGC, which contained 38,691 cases and 186,843 controls. GWAS summary statistics for ADHD subtypes were obtained from iPSYCH, which included childhood (Ncase = 14,878), late‐diagnosed (Ncase = 6961), and persistent ADHD (Ncase = 1473), alongside 38,303 controls (Rajagopal et al. 2022).

To mitigate biases from integration artifacts, batch effects, and population mismatches, we included only studies with documented quality control and explicit confounder adjustment (e.g., population structure, technical variation) in primary analyses. The datasets were restricted to individuals of European ancestry to ensure genetic homogeneity and minimize population stratification across multi‐omic integration. More detailed information about the eQTL and GWAS datasets is summarized in Table S1.

### 2.2 MR Analysis

MR analysis must meet three core assumptions: the relevance assumption, the independence assumption, and the exclusion restriction assumption (Davey Smith and Ebrahim 2003). To obtain eligible instrumental variables (IVs), we first selected exposure‐related single‐nucleotide polymorphisms (SNPs). For cis‐eQTLs, we set the screening threshold to a false discovery rate (FDR) < 0.05 and performed a clumping procedure with a window size of 10 MB and an *r* ^2^ threshold of 0.1. The selection of MR parameters balances statistical rigor with the unique challenges of our analysis. For linkage disequilibrium (LD) clumping, the *r^2^* < 0.1 criterion was informed by literature demonstrating that strict LD‐pruning risks discarding biologically relevant cis‐variants due to the localized LD structure of gene neighborhoods (Gkatzionis et al. 2023). This less aggressive pruning retains variants functionally linked to gene expression while reducing redundancy. The FDR criterion (FDR < 0.05) is widely adopted in transcriptomic and proteomic MR analyses. Unlike the more conservative Bonferroni correction, FDR effectively controls FDRs while retaining greater statistical power, and is particularly suited to cis‐eQTL analyses testing fewer variants (typically 1 Mb upstream/downstream) compared to genome‐wide association scans (Su et al. 2023; Storm et al. 2021). These thresholds optimize statistical power by retaining more IVs to increase the proportion of exposure variance explained (*R* ^2^), especially critical for single‐cell datasets with smaller sample sizes (Brion et al. 2013).

For genetic variants associated with CTP, we set a tighter screening threshold (*p* < 5 × 10^−6^) and clumping procedure (a window size of 10Mb and an *r* ^2^ threshold of 0.001). In addition, all exposure‐related SNPs were not associated with the outcomes (*p* > 5 × 10^−8^) and passed the Steiger filtering test. Finally, to ensure sufficient statistical strength of the IVs, SNPs with *F* statistics < 10 were excluded.

We subsequently performed data harmonization, and SNPs with palindromic alleles with intermediate allele frequencies > 0.42 were removed. The inverse variance weighted (IVW) method was the primary MR analysis method for exposures with multiple IVs. For exposures with only one IV, the Wald ratio method was used. For exposures with three or more IVs, we also employed the weighted median and MR‒Egger regression methods for sensitivity analyses. If the MR estimates generated by these methods have the same direction, the results are considered robust. Furthermore, MR‒Egger regression can also assess potential directional pleiotropy by examining the intercept term (Bowden et al. 2015). Cochran's *Q* test was used to evaluate the heterogeneity of IVW analyses; if there was no heterogeneity (*p* > 0.05 for the test of Cochran's Q), we chose fixed‐effects IVW as the main method; otherwise, the random‐effects IVW method was applied. The multiple correction level for the single‐cell eQTL MR analysis result was set at *p* less than the Bonferroni‐corrected threshold (0.05/the number of genes used for MR analysis in a given cell type) (Table S2). MR analysis was performed via the TwoSampleMR (Version: 0.6.5) (Hemani et al. 2018) and ieugwasr (Version: 1.0.1) packages in R software (Version 4.2.1).

### 2.3 Bayesian Colocalization Analysis

The coloc R package (Version: 5.2.3) (Giambartolomei et al. 2014) was used to perform genetic colocalization analysis of single‐cell cis‐eQTLs and ADHD GWAS under a single causal variant assumption to determine whether they share genetic causal variants. On the basis of the predefined prior probabilities (*P*_1_ = 1 × 10^−4^, *P*_2_ = 1 × 10^−4^, *P*_12_ = 1 × 10^−5^), the analysis provided the posterior probabilities for five hypotheses regarding whether a single variable is shared between two traits: (i) PPH0: the probability that neither trait has a genetic association in the region; (ii) PPH1: the probability that only Trait 1 has a genetic association in the region; (iii) PPH2: the probability that only Trait 2 is genetically associated in the region; (iv) PPH3: the probability that both traits are associated but with different causal variants; and (v) PPH4: the probability that both traits are associated and share a causal variant. In this study, a posterior probability (PPH4) of ≥ 80% indicates strong evidence of colocalization between single‐cell cis‐eQTLs and ADHD GWAS. A PPH4 value in the range of 50% ≤ PPH4< 80% indicates moderate evidence of colocalization between single‐cell cis‐eQTLs and ADHD GWAS.

### 2.4 Bioinformatics Analysis

For the causal genes supported by MR and Bayesian colocalization analyses, we conducted a series of bioinformatics analyses. Kyoto Encyclopedia of Genes and Genomes (KEGG), Gene Ontology (GO), and GWAS catalog library analyses were carried out using the Enrichr web tool (https://maayanlab.cloud/Enrichr/). The PPI network was constructed based on the STRING database (https://string‐db.org), with a minimum required interaction score set to 0.25. In addition, we investigated the expression of these identified causal genes in different brain cell types (including excitatory and inhibitory neurons, glial cells, and cell types related to development, etc.). The Wilcoxon Rank Sum test was used to analyze whether the expression of target genes was enriched in specific cell types, with a threshold set for FDR‐corrected *p* < 0.05 and log_2_ (fold‐change) > 1. We also utilized the Cortical Development Expression Viewer (CoDEx) data portal (http://solo.bmap.ucla.edu/shiny/webapp/) to explore the single‐cell gene expression pattern of the developing human cortex.

Lastly, we used expression perturbation data from the CMap database (Subramanian et al. 2017) (https://clue.io) to screen for small molecule compounds with highly similar or opposite expression signatures to the identified causal genes (enrichment scores > 90 or < −90). If the overexpression or knock‐down reagent of a gene is similar or opposite to the gene expression signature induced by the treatment of a compound, it indicates that the compound may promote or repress the expression of the gene. Combining the direction of MR effect estimates of gene expression and ADHD risk, we can screen small molecule compounds as drug candidates, thus providing drug repurposing opportunities for ADHD patients. We then performed molecular docking to test the targeting ability of these compounds. The 3D structures of the proteins were obtained from the PDB (https://www.rcsb.org/pdb) or AlphaFold (https://alphafold.ebi.ac.uk) databases, and the 3D structures of the compounds were downloaded from the PubChem database (https://pubchem.ncbi.nlm.nih.gov/). Molecular docking studies were performed using AutoDock Vina (Version 1.1.2) and AutoDock Tools (Version 1.5.6) (Trott and Olson 2010). Structure visualization was done with PyMOL (The PyMOL Molecular Graphics System, Version 2.2.0, Schrödinger, LLC.).

## 3 Results

### 3.1 Single‐Cell eQTL MR and Bayesian Colocalization Analyses Identified Causal Genes Affecting ADHD Risk in Specific Brain Cell Types

**FIGURE 2:**
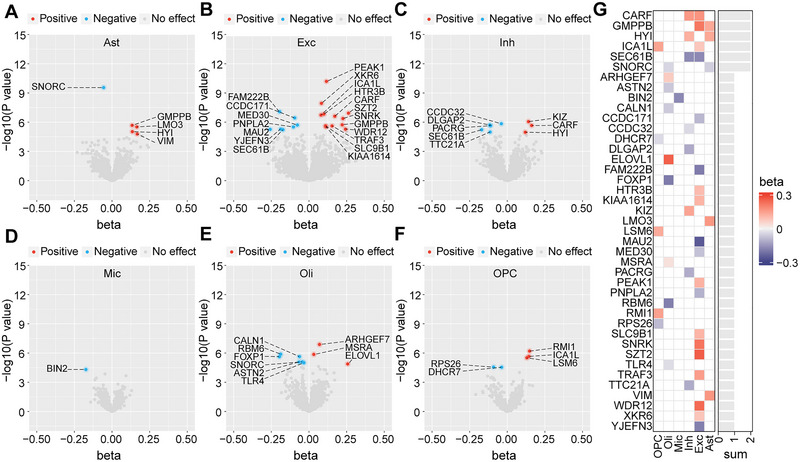
**Results of single‐cell eQTL MR analysis for causal effects of genes on ADHD. A–F,** Volcano plots showing the MR estimates of (**A**) astrocytes, (**B**) excitatory neurons, (**C**) inhibitory neurons, (**D**) microglia, (**E**) oligodendrocytes, and (**F**) oligodendrocyte progenitor cells on ADHD. **G,** Heat map summarizing the significant results (after Bonferroni correction) of MR analysis in six brain cell types.

Single‐cell eQTL MR analysis identified a total of 47 gene‒cell‐outcome associations that were significant after Bonferroni correction (involving 41 unique genes), of which 5 genes in astrocytes, 19 genes in excitatory neurons, 8 genes in inhibitory neurons, 1 gene in microglia, 9 genes in oligodendrocytes, and 5 genes in oligodendrocyte precursor cells were associated with ADHD risk (Figure 2A–F). Notably, CARF, GMPPB, HYI, ICA1L, SEC61B, and SNORC showed cross‐cell effects in their associations with ADHD risk, whereas other associations were cell‐specific (Figure 2G). Sensitivity analyses indicated that the associations of ASTN2 in oligodendrocytes, DLGAP2 in inhibitory neurons, and MSRA in oligodendrocytes with ADHD risk were not entirely consistent in the effect estimation direction across the three MR methods (Table S3). No potential horizontal pleiotropy or heterogeneity was found in the associations supported by MR analysis (Table S4).

### 3.2 Comparison of the Results From Single‐Cell eQTL MR and Bulk Tissue eQTL MR Analyses

**FIGURE 3:**
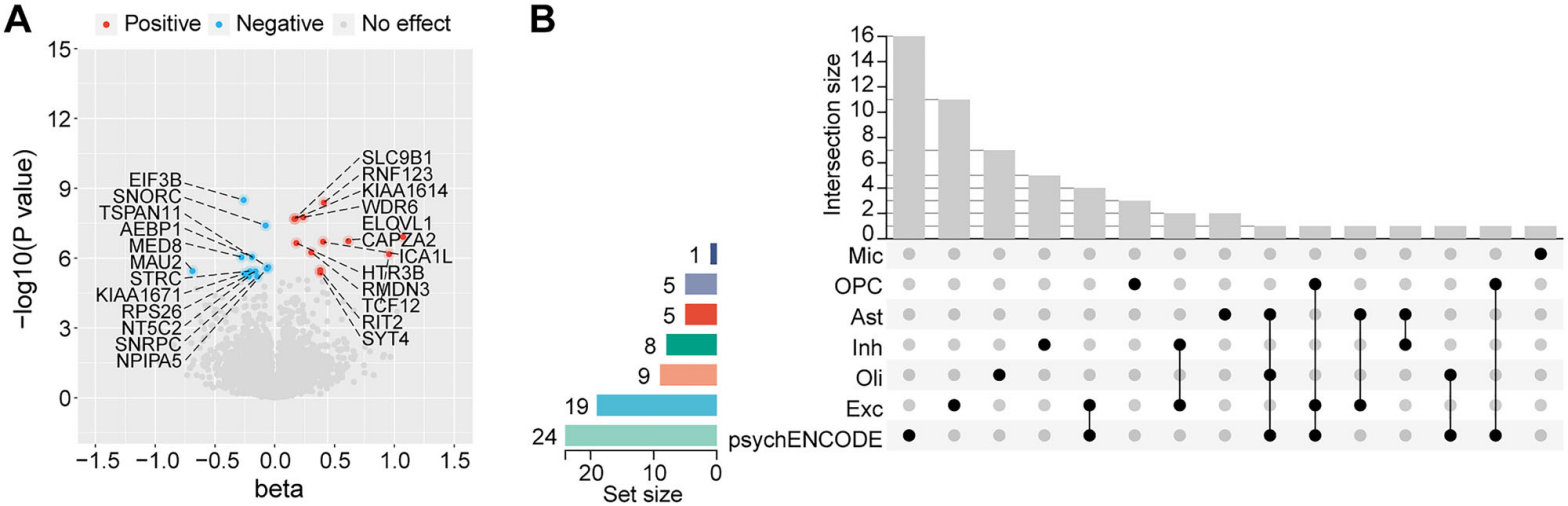
**Results of bulk tissue eQTL MR analysis. A,** Volcano plots showing the results of bulk tissue eQTL MR analysis for causal effects of genes on ADHD. **B,** UpSet plot showing causal genes overlapping across different cells or tissues.

For genes with cis‐eQTLs at the bulk tissue level, we also conducted MR analysis. After Bonferroni correction, bulk tissue eQTL MR analysis identified 24 genes significantly associated with ADHD risk in the prefrontal cortex (Figure 3A and Table S5). Upon comparison, we found that 8 genes were significantly associated with ADHD risk across different levels of eQTL MR analysis, whereas 33 genes showed significant causal associations exclusively in single‐cell eQTL MR analysis (Figure 3B). Spearman correlation analysis showed that there were correlations between MR estimates of the same genes in bulk brain tissue and different brain cell types and that most of these correlations were enhanced as the *p* value screening thresholds (no threshold, *p* < 0.5, and *p* < 0.25) for the MR results became stringent, except for the correlation between MR estimates of genes in OPC and in bulk tissue, which became non‐significant (Figure S1).

### 3.3 Assessing the Causal Effects of Identified Genes on ADHD Subtypes

**FIGURE 4:**
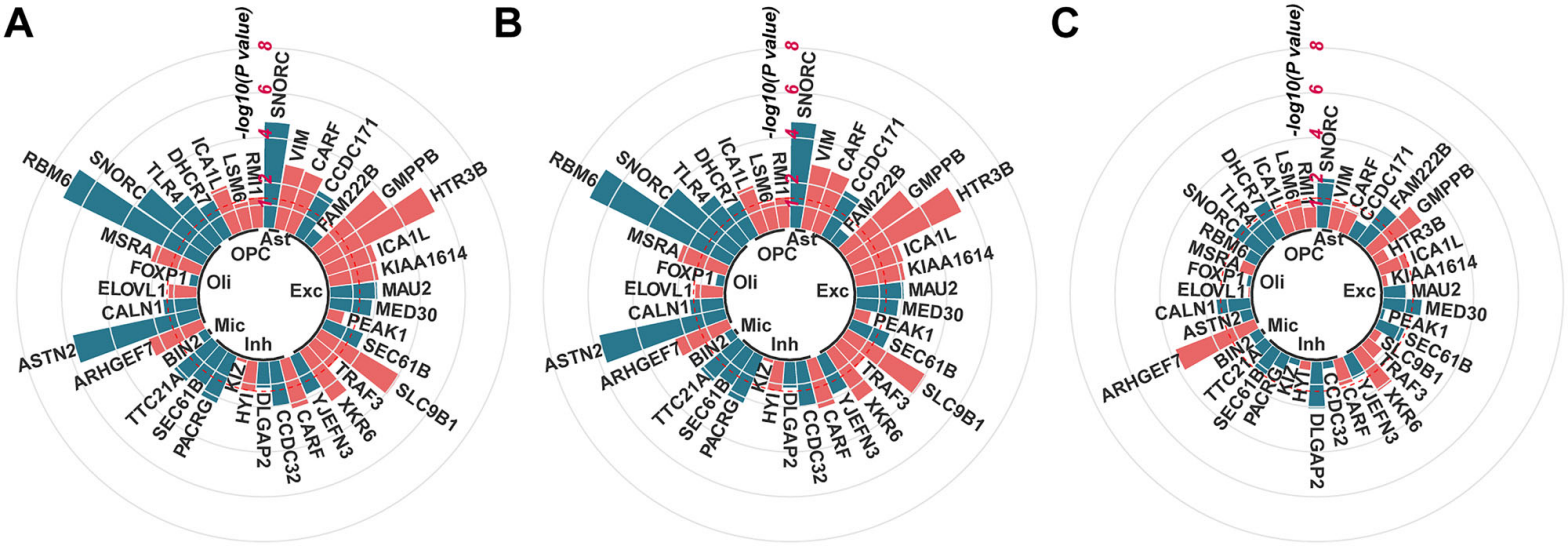
**Causal effects of identified genes on ADHD subtypes. A–C,** Results of single‐cell eQTL MR analysis for causal effects of identified genes on **(A)** childhood, **(B)** late‐diagnosed, and **(C)** persistent ADHD. The red dashed line indicates the threshold of significance (*p <* 0.05).

We also investigated whether there was an association between identified causal genes and the risk of specific ADHD subtypes (Table S6–S8). A total of 35 genes had eligible IVs for MR analysis. We found that most risk genes had similar causal effects across ADHD subtypes (Figure 4A–C). Following Bonferroni correction, 22 gene‒cell associations remained significant in childhood ADHD, 15 in late‐diagnosed ADHD, and 3 in persistent ADHD. Notably, CARF in astrocytes; CCDC171, PEAK1, and SLC9B1 in excitatory neurons; DLGAP2 in inhibitory neurons; and ARHGEF7 and TLR4 in oligodendrocytes were all strongly associated with childhood ADHD. In addition, SNORC in astrocytes, HTR3B in excitatory neurons, ASTN2, and RBM6 in oligodendrocytes showed a stronger association with late‐diagnosed ADHD. ARHGEF7 in oligodendrocytes showed a stronger association with the risk of persistent ADHD, while most genes showed no significant association with this subtype.

### 3.4 Bayesian Colocalization Analysis

As an important supplementary analysis to MR studies, associations supported by colocalization analysis have a higher priority for druggability, which facilitates the identification and validation of drug targets (Zuber et al. 2022). Among the 47 MR‐supported associations, 12 showed strong colocalization evidence (PPH4 ≥ 80%), 16 showed moderate colocalization evidence (PPH4 ≥ 50%), and 19 showed weaker colocalization evidence (PPH4< 50%) (Table 1). A total of 24 unique genes had strong or moderate colocalization evidence with ADHD, among which CARF, GMPPB, ICA1L, and SEC61B had overlapping loci with ADHD across multiple brain cell types.

**TABLE 1:** Results of colocalization analysis of single‐cell cis‐eQTLs and ADHD GWAS.

Gene	Cell type	PPH0	PPH1	PPH2	PPH3	PPH4	Colocalization evidence
PNPLA2	Exc	9.91E‐28	8.51E‐27	0.00102	0.00774	0.991	Strong
RMI1	OPC	0.0000269	0.000383	0.00103	0.0137	0.985	Strong
SEC61B	Exc	1.52E‐15	4.45E‐15	0.00817	0.0229	0.969	Strong
CALN1	Oli	3.46E‐43	4.19E‐42	0.00307	0.0362	0.961	Strong
PEAK1	Exc	1.22E‐33	5.34E‐33	0.0112	0.0479	0.941	Strong
SEC61B	Inh	0.000151	0.000442	0.0175	0.05	0.932	Strong
VIM	Ast	0.0000144	0.0000944	0.0122	0.0796	0.908	Strong
MAU2	Exc	0.0000117	0.0000568	0.0207	0.0994	0.88	Strong
RPS26	OPC	4.29E‐11	1.25E‐10	0.0398	0.115	0.845	Strong
BIN2	Mic	0.0000954	0.000287	0.0458	0.137	0.817	Strong
TRAF3	Exc	0.000000018	0.000000259	0.0124	0.178	0.809	Strong
FAM222B	Exc	2.06E‐23	2.16E‐23	0.0964	0.1	0.803	Strong
CARF	Inh	0.000000165	0.0000175	0.00187	0.199	0.8	Moderate
ICA1L	OPC	0.00000866	0.000924	0.00188	0.2	0.798	Moderate
LMO3	Ast	0.00447	0.00131	0.154	0.0442	0.796	Moderate
ICA1L	Exc	1.28E‐60	1.37E‐58	0.00191	0.202	0.796	Moderate
CARF	Exc	9.32E‐10	9.94E‐08	0.00192	0.204	0.794	Moderate
WDR12	Exc	0.00000014	0.000015	0.00195	0.207	0.791	Moderate
GMPPB	Exc	0.000153	0.0222	0.00129	0.186	0.79	Moderate
GMPPB	Ast	0.00000699	0.00101	0.00185	0.267	0.73	Moderate
SLC9B1	Exc	4.03E‐10	8.37E‐09	0.0129	0.268	0.719	Moderate
LSM6	OPC	0.00000159	0.00229	0.000207	0.299	0.699	Moderate
SNRK	Exc	0.000000407	0.000842	0.000157	0.324	0.675	Moderate
YJEFN3	Exc	0.00000114	0.00000547	0.0631	0.303	0.634	Moderate
DHCR7	OPC	7.06E‐57	2.24E‐57	0.281	0.0887	0.63	Moderate
ARHGEF7	Oli	5.4E‐34	5.4E‐34	0.191	0.191	0.618	Moderate
CCDC171	Exc	9.71E‐39	2.45E‐38	0.118	0.298	0.584	Moderate
PACRG	Inh	4.16E‐09	3.38E‐09	0.233	0.189	0.578	Moderate
SNORC	Ast	1.02E‐95	2.39E‐94	0.0223	0.521	0.457	Weak
MED30	Exc	5.23E‐43	5.74E‐44	0.497	0.054	0.449	Weak
SNORC	Oli	3.11E‐45	7.27E‐44	0.0234	0.546	0.43	Weak
RBM6	Oli	0.0000109	0.00158	0.00438	0.633	0.361	Weak
FOXP1	Oli	3.58E‐10	0.000000823	0.000294	0.675	0.325	Weak
XKR6	Exc	8.74E‐27	3.19E‐27	0.526	0.192	0.282	Weak
TTC21A	Inh	0.00515	0.000743	0.631	0.0908	0.272	Weak
HTR3B	Exc	7.13E‐10	3.36E‐09	0.127	0.601	0.272	Weak
KIAA1614	Exc	0.000000037	9.7E‐09	0.714	0.187	0.0987	Weak
MSRA	Oli	1.92E‐44	1.32E‐44	0.565	0.388	0.0474	Weak
CCDC32	Inh	3.05E‐93	2.91E‐93	0.497	0.473	0.03	Weak
DLGAP2	Inh	6.72E‐10	1.43E‐09	0.31	0.662	0.0281	Weak
TLR4	Oli	1.19E‐98	1.38E‐97	0.0783	0.91	0.0114	Weak
ASTN2	Oli	1.18E‐12	1.37E‐11	0.0786	0.919	0.00254	Weak
HYI	Ast	4.41E‐13	0.000121	3.62E‐09	0.997	0.00246	Weak
ELOVL1	Oli	9.86E‐11	0.0271	3.52E‐09	0.971	0.00235	Weak
HYI	Inh	4.92E‐12	0.00136	3.63E‐09	0.998	0.000203	Weak
SZT2	Exc	3.47E‐17	9.55E‐09	3.63E‐09	1	0.00002	Weak
KIZ	Inh	1.25E‐11	0.0000209	0.000000601	1	0.00000277	Weak

### 3.5 Causal Estimates of CTP in the Prefrontal Cortex on ADHD

**FIGURE 5:**
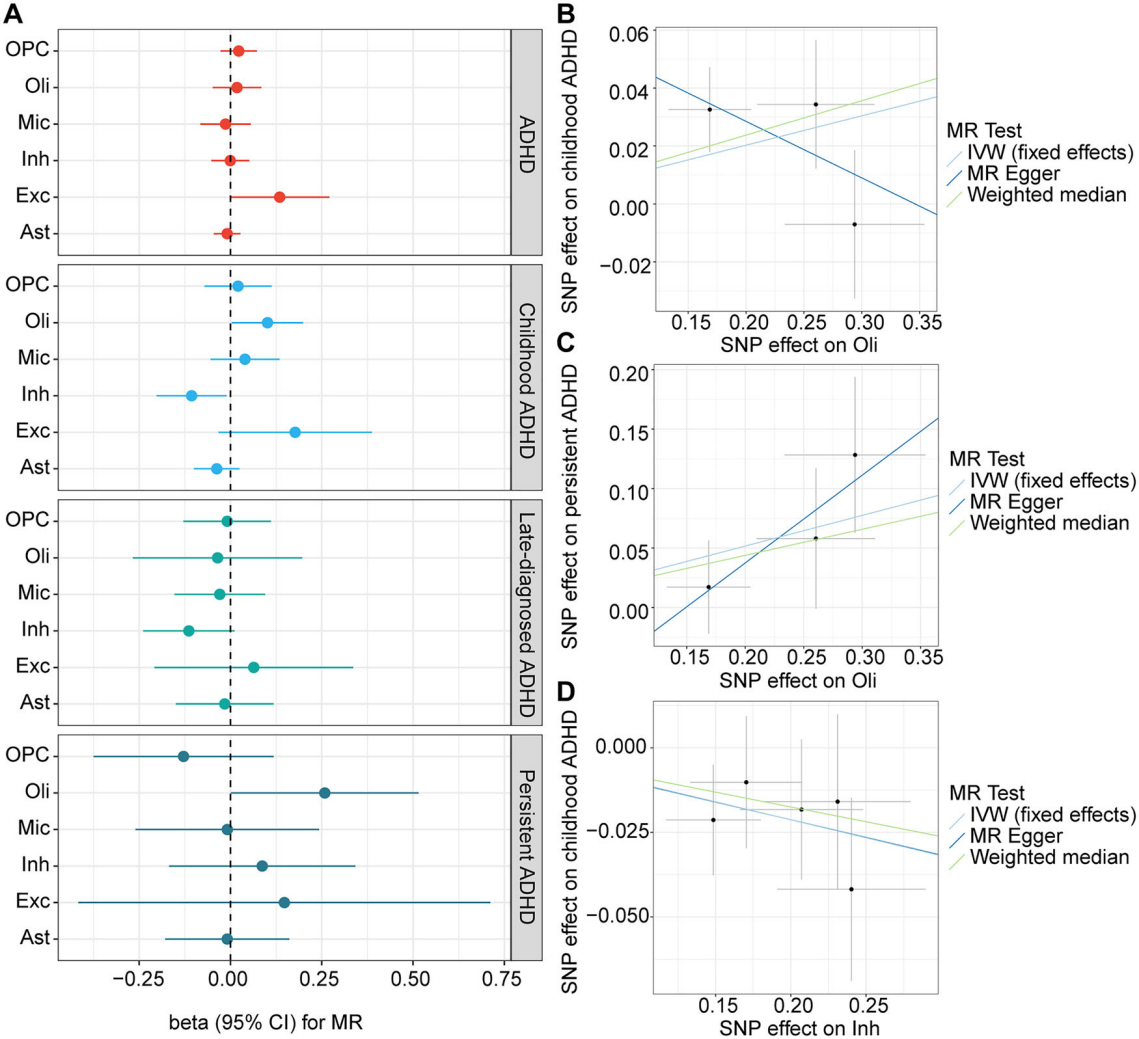
**Results of MR analysis for causal effects of CTP in the prefrontal cortex on ADHD outcomes. A,** Forest plot showing the causal effects of CTP on ADHD outcomes. **B–D,** Sensitivity analyses employing three MR methods (IVW, MR Egger and Weighted median) to evaluate causal effects of the proportion of oligodendrocytes on **(B)** childhood and **(C)** persistent ADHD and of the proportion of inhibitory neurons on **(D)** childhood ADHD. Each point in the scatter plots represents a single nucleotide polymorphism (SNP). The slope of the lines in scatter plots reflects the effect size of the relationship between CTP and ADHD risk. Each method is visually distinguished by a unique line (as labeled in each panel's legend).

We further evaluated the causal estimates of CTP in the prefrontal cortex on ADHD using MR analysis. The results indicated a suggestive causal association between an increased proportion of oligodendrocytes and an increased risk of childhood and persistent ADHD (*β* = 0.101, SE = 0.0498, *p* = 0.042; *β* = 0.258, SE = 0.131, *p* = 0.049, respectively). Conversely, a reduced proportion of inhibitory neurons was suggestively linked to an increased risk of childhood ADHD (*β* = −0.106, SE = 0.0489, *p* = 0.030) (Figure 5A). Sensitivity analyses revealed that the association between oligodendrocyte proportion and childhood ADHD risk was not entirely consistent across the three MR methods in the direction of effect estimates, whereas the associations between oligodendrocyte proportion and persistent ADHD risk, and between inhibitory neuron proportion and childhood ADHD risk, were robust (Figure 5B–D and Table S9).

### 3.6 Bioinformatics Analysis

**FIGURE 6:**
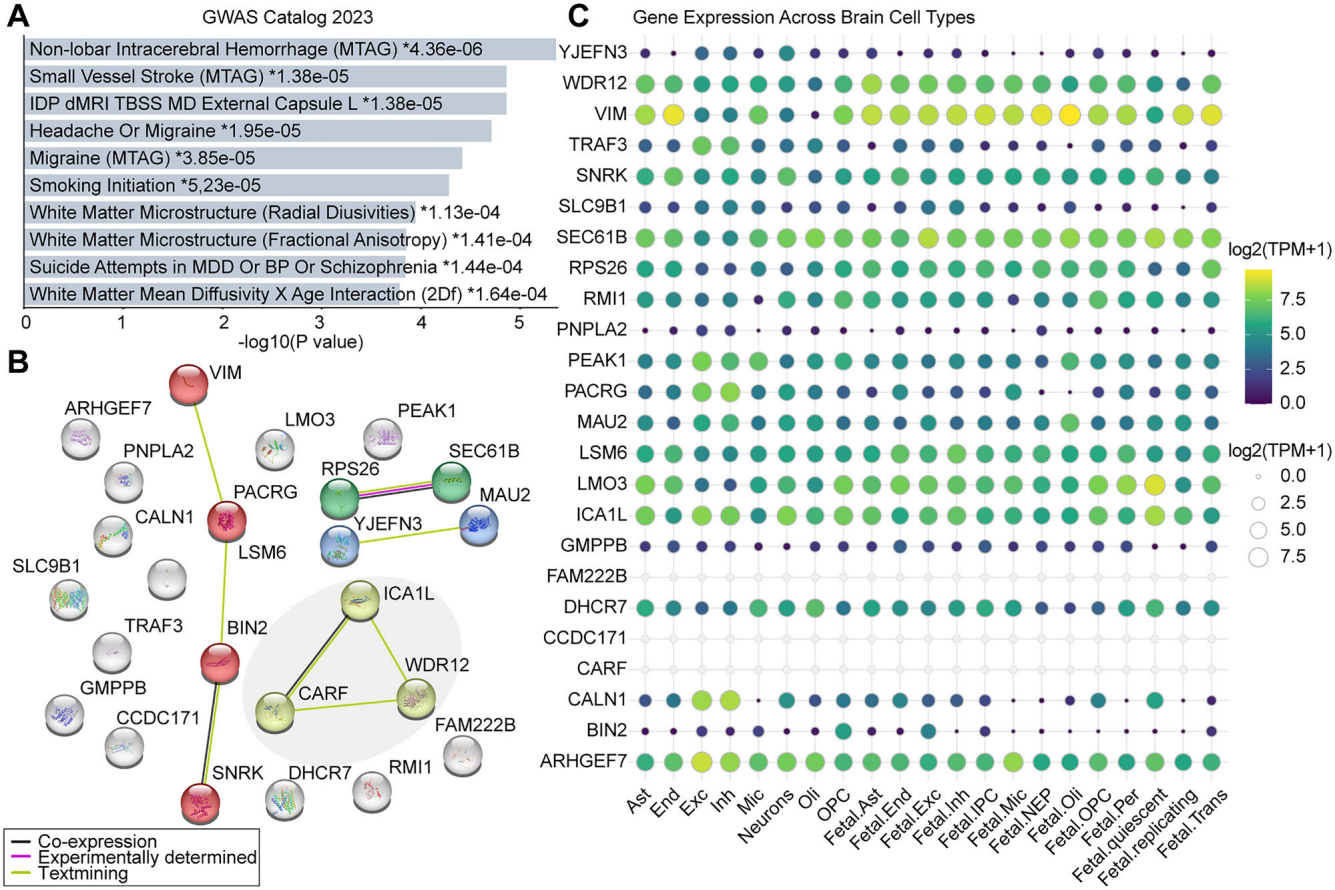
**Results of bioinformatic analyses. A,** The top 10 enriched terms of the GWAS catalog library of the identified causal genes. **B,** PPI network of the identified causal genes. **C,** Gene expression across different brain cell types from the prefrontal cortex. Asterisks indicate FDR‐corrected *p* < 0.05.

**FIGURE 7:**
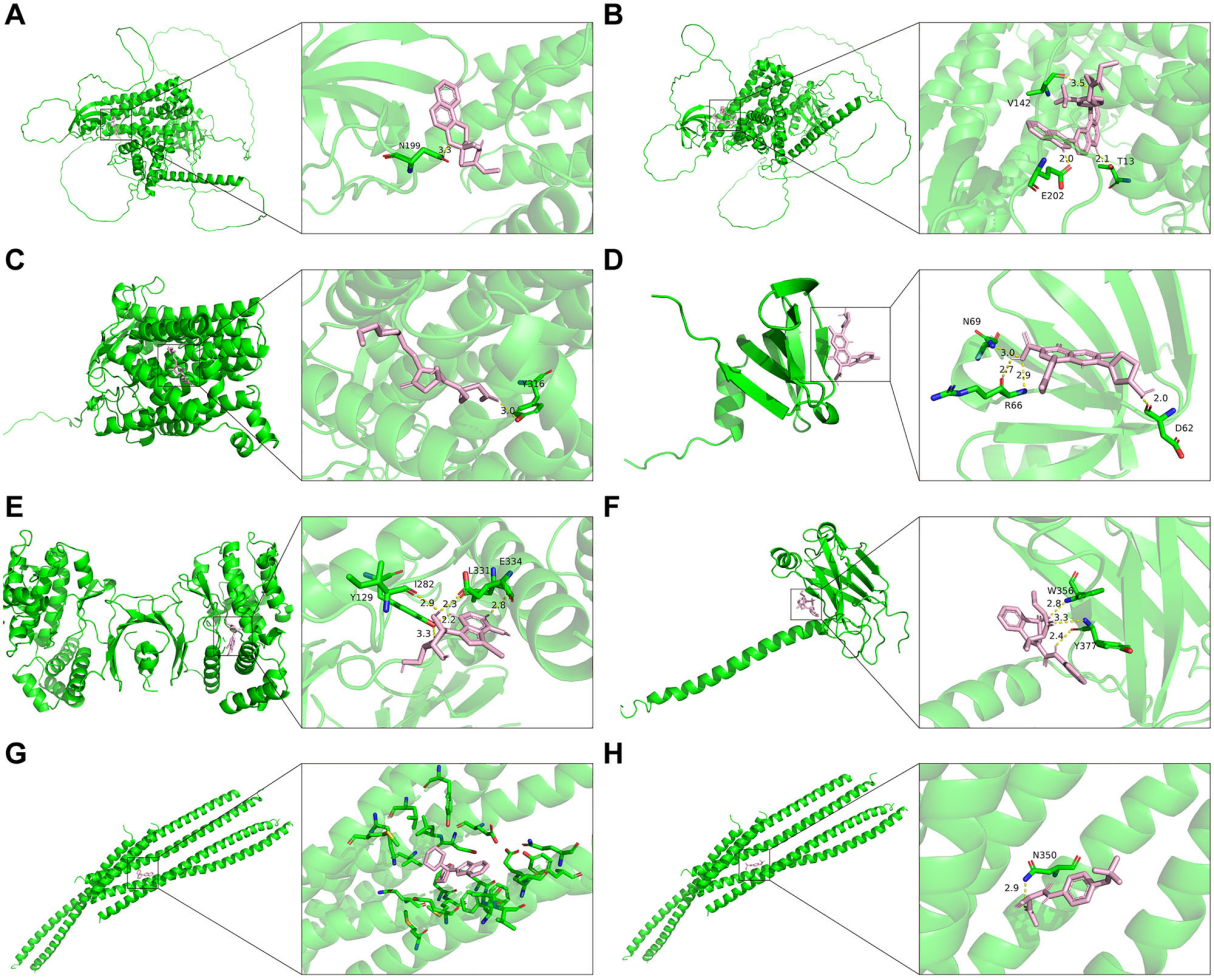
**Structure visualization of molecular docking**. **(A)** ARHGEF7 and DMP‐543; **(B)** ARHGEF7 and torin‐1; **(C)** DHCR7 and prostaglandin; **(D)** LSM6 and tosufloxacin; **(E)** SNRK and 5‐iodotubercidin; **(F)** TRAF3 and ampiroxicam; **(G)** VIM and CS‐110266; **(H) **VIM and LFM‐A12.

We then conducted a series of bioinformatics analyses of 24 risk genes supported by single‐cell eQTL MR and Bayesian colocalization analyses. KEGG and GO analyses indicated that these genes were not significantly enriched in any particular pathological pathway (FDR > 0.05) (Figure S2), whereas the GWAS catalog library enrichment analysis identified associations of these genes with non‐lobar intracerebral hemorrhage, small vessel stroke, headache or migraine, white matter microstructure (radial diffusivities or fractional anisotropy), and suicide attempts in major depressive disorder, bipolar disorder, or schizophrenia (Figure 6A). In addition, Figure 6B illustrates the interactions within a 24‐node, 8‐edge PPI network. CARF, ICA1L, and WDR12 were found to interact with each other and were involved in most of the enrichment analysis items. Twenty‐one genes (excluding CARF, CCDC171, and FAM222B) were detected to be expressed in brain cells, with 16 genes showing enriched expression in specific brain cell types (Figure 6C and Table S10). We observed several cell‐specific gene expressions that corresponded to the associations supported by MR: DHCR7 was enriched in oligodendrocytes, LMO3, and VIM in developing or mature astrocytes, PNPLA2, PEAK1, and TRAF3 in excitatory neurons, RPS26 in oligodendrocyte precursor cell lineages, and SLC9B1 in developing excitatory and inhibitory neurons. We also explored the expression patterns of these genes using single‐cell expression data from the developing human cortex. ARHGEF7, RPS26, and SEC61B were found to be widely expressed across different brain cell types, whereas CALN1, CCDC171, and LMO3 were specifically expressed in excitatory deep layer 1, and VIM was specifically expressed in radial glia (Figure S3). Finally, we retrieved the expression perturbation data for six genes from the CMap database and screened for compounds with highly similar or opposite expression signatures to these genes (Table S11). The compounds with the highest enrichment scores were selected for molecular docking to assess the affinity of the drug candidates for their targets. A total of 8 protein‒ligand dockings were involved, all of which showed good binding activity (binding energy less than −5 kcal/mol) (Table 2). Except for the binding of VIM to CS‐110266, the remaining docking pairs exhibited stable binding via hydrogen bonds (Figure 7).

**TABLE 2:** Results of CMap analysis and molecular docking.

Gene	Compound	Perturbagen type	CMap enrichment score	Binding energy (kcal/mol)	PDB	AlphaFold
ARHGEF7	Selinidin	Gene over‐expression	−97.3	−7.5	—	Q14155
ARHGEF7	Torin‐1	Gene knock‐down	99.92	−8.8	—	Q14155
DHCR7	Prostaglandin	Gene knock‐down	−96.01	−7.7	—	Q9UBM7
LSM6	Tosufloxacin	Gene knock‐down	99.92	−6.6	—	P62312
SNRK	5‐iodotubercidin	Gene knock‐down	99.58	−6.5	5YKS	—
TRAF3	Ampiroxicam	Gene knock‐down	99.89	−6.9	1FLK	—
VIM	CS‐110266	Gene knock‐down	99.55	−7.9	1GK4	—
VIM	LFM‐A12	Gene over‐expression	−93.31	−6.9	1GK4	—

## 4 Discussion

This research first evaluated the associations between the genetic regulation of specific brain cell types in the prefrontal cortex and the risk of ADHD. Single‐cell eQTL MR analyses supported 47 gene‒cell‐outcome causal associations, which were cell‐ and subtype‐specific. Subsequent Bayesian colocalization analysis supported 28 associations, involving 24 unique risk genes. In addition, the proportions of inhibitory neurons and oligodendrocytes in the prefrontal cortex were linked to the risk of specific subtypes of ADHD. Finally, we delved into these risk genes through a series of bioinformatics analyses, providing new insights into the neurobiology of ADHD and potential therapeutic targets.

Identifying disease‐risk genes from a genetic perspective is a common strategy; however, exploring the biological mechanisms related to SNPs may become more complex. This is because SNPs may have tissue or cell‐type‐specific effects on complex traits (Fujita et al. 2024; GTEx Consortium 2020). Compared with the results from bulk brain tissue, most causal associations supported by single‐cell eQTL MR were newly identified, which reflect brain cell‐specific regulation of ADHD risk. In a recently published MR study that examined brain, cerebrospinal fluid, and plasma proteins linked to ADHD risk, it was found that the expression of GMPPB, HYI, and ICA1L proteins in the DLPFC was also significantly associated with ADHD risk (C. Zhang et al. 2024). Notably, most genes showed causal effects predominantly in specific brain cell types, especially excitatory neurons. This suggests that gene expression related to excitatory neurons may be particularly relevant to ADHD among the cell types analyzed. In fact, excitatory neurons are the most abundant cell type in the DLPFC (Ma et al. 2022; Maitra et al. 2023). Their extensive connectivity and role in neural signal transduction are critical for both normal function and pathological alterations in the DLPFC. Furthermore, numerous studies have reported abnormalities in the excitatory neurotransmitter system in patients with ADHD. Specifically, dysregulation of the glutamatergic system, which is primarily mediated by excitatory neurons, has been well documented (Yates 2024; Choi et al. 2024; Huang et al. 2019). These findings collectively suggest that excitatory neurons play a key role in the pathophysiology of ADHD. However, this pattern may also reflect differences in the number of cis‐eQTLs across various brain cell types, as excitatory neurons have the highest number of genes available for MR analysis (Fujita et al. 2024; Wei et al. 2022). Large‐scale GWAS has indicated genetic architecture differences among ADHD subtypes (Rajagopal et al. 2022). Correspondingly, specific genes showed suggestive associations with certain subtypes, implying potential subtype‐specificity in the brain cell‐mediated regulation of ADHD risk. These preliminary findings highlight the need to investigate differentiated therapeutic strategies for ADHD subtypes in future studies.

Bayesian colocalization analysis identified 28 robust associations with colocalization evidence, involving 24 unique genes. This suggests that they have a higher priority for druggability as potential brain cell‐specific therapeutic targets. Reviewing the druggable gene list reported by Finan et al. (2017), we found that DHCR7, PEAK1, and SNRK are effective targets for approved small molecules, biotherapeutic drugs, and clinical‐phase drug candidates. Although not yet used for ADHD, studies have linked them to the pathogenesis of ADHD. For example, mutations in DHCR7 can block cholesterol synthesis, causing 7‐DHC accumulation and Smith–Lemli–Opitz syndrome (SLOS), where ADHD is a typical symptom (Miyazaki et al. 2024). To further identify the druggability of these targets, we used CMap analysis to examine the gene‒compound‒disease associations, integrating with molecular docking data to provide ADHD drug repurposing opportunities. However, further pharmacological studies in the field of ADHD are yet to be carried out. Thus, the translation of CMap analysis‐derived results into clinical practice needs to be carefully considered (Qu and Rajpal 2012).

The integration with genetic data highlights the contribution of genetics to changes in CTP (Yap et al. 2024). Using these data, we found that the proportion of inhibitory neurons and oligodendrocytes in the prefrontal cortex was linked to the risk of specific ADHD subtypes. Patients with ADHD exhibit an imbalance of excitatory/inhibitory neurotransmitters. Gamma‐aminobutyric acid (GABA), released by inhibitory neurons, is a key pathophysiological neurotransmitter in ADHD (Naaijen et al. 2017). Reduced GABA signaling in the prefrontal cortex is associated with ADHD in clinical populations (Ferranti et al. 2024). In healthy individuals, increased GABA levels are related to improvements in working memory and attention tasks (Yoon et al. 2016). These conclusions support our findings to a certain extent. However, research on the relationship between oligodendrocytes and ADHD is currently limited. Patients with ADHD show abnormalities in white matter microstructure (Aoki et al. 2017). Notably, oligodendrocytes, the major glial cells in the brain's white matter, may contribute to ADHD pathogenesis (Bennett and Lagopoulos 2015). Subsequent gene enrichment analyses revealed the interactions between ICA1L, CARF, and WDR12 related to white matter microstructure (Jian et al. 2018). These findings provide insights into the potential pathogenic mechanisms of these genes. Gene expression analysis showed that DHCR7, LMO3, PEAK1, PNPLA2, TRAF3, RPS26, SLC9B1, and VIM were enriched in specific brain cell types, aligning with MR‐supported associations and further enhancing the robustness of our conclusions.

Although our study has made certain advancements in the field of ADHD, there are still some limitations in result interpretation and generalizability. First, the data we used are derived primarily from European populations. Genetic data from different ethnic populations can increase the universality of MR studies and uncover population‐specific causal effects (Weng et al. 2023; Hu et al. 2023).

Therefore, future validation in different ethnic populations is necessary. Second, due to the cost of single‐cell sequencing, single‐cell eQTLs have not been identified in a large population, limiting the discovery of cell‐specific genetic variants and hindering comprehensive heterogeneity and pleiotropy testing (J. Zhang and Zhao 2023). Notably, emerging evidence shows that numerous eQTLs exhibit functionality exclusively in specific cell subtypes (Emani et al. 2024). In addition, brain cell subtypes are increasingly recognized for their distinct regulatory roles in neurodevelopment. For example, a recent study demonstrated that dysfunction in parvalbumin‐positive (PV+) neurons, a major inhibitory neuron subtype, could drive core behavioral phenotypes of ADHD (An et al. 2025). Our analysis failed to resolve functionally specialized subpopulations. This may have obscured subtype‐specific effects, highlighting the need for targeted single‐cell eQTL MR analyses in defined cell subclasses. Third, while our MR framework effectively identifies genetically predicted associations, it primarily reflects lifetime genetic liability rather than acute, dynamic gene expression changes in specific cellular contexts. Fourth, reliance on summary‐level data rather than individual‐level raw data in our approach restricts the ability to perform fine‐grained control for confounders in multi‐omic integration.

Our strategies depended on the primary studies’ quality control and ancestry harmonization across datasets. This trade‐off highlights the challenges of leveraging publicly accessible multi‐omic resources. Lastly, although we identified several brain cell‐specific risk genes, mechanistic validation in laboratory models or clinical cohorts is essential. Future research on their cell‐specific expression regulation will provide a more comprehensive understanding of ADHD pathogenesis and drug development.

## 5 Conclusion

To conclude, using MR, Bayesian colocalization, and bioinformatics analyses, our study revealed causal associations between CTP in the prefrontal cortex and their regulation of gene expression with the risk of ADHD. These findings clarify the neurobiological mechanisms of ADHD and highlight potential targets for developing new treatments.

## Author Contributions

**Jiawei Gui**: formal analysis, investigation, methodology, software, visualization, writing – original draft. **Ziyi Xia**: writing – review and editing, investigation. **Keqi Wan**: data curation, writing – review and editing. **Xiangli Dong**: resources, writing – review and editing. **Weiming Sun**: conceptualization, funding acquisition, methodology, project administration, supervision.

## Ethics Statement

The relevant institutional review boards approved the data sources; no additional ethical approval was needed for this study.

## Consent

The authors have nothing to report.

## Conflicts of Interest

The authors declare no conflicts of interest.

## Peer Review

The peer review history for this article is available at https://publons.com/publon/10.1002/brb3.70664

References

## Supporting information




**Supplementary Figures**: brb370664‐sup‐0001‐Figures.docx

**Supplementary Tables**: brb370664‐sup‐0002‐Tables.xlsx

## Data Availability

All the data sources used in this study can be acquired from the original studies. Single‐cell cis‐eQTLs are available at Synapse (https://doi.org/10.7303/syn52335732). The GWAS summary statistics for ADHD and its subtypes can be downloaded from the PGC website (https://www.med.unc.edu/pgc/download‐results/) and the iPSYCH website (https://ipsych.dk/en/research/downloads/). GWAS summary statistics for brain CTPs are available at Zenodo (https://doi.org/10.5281/zenodo.7604233). The merged PsychENCODE and GTEx brain cell gene expression matrix for the prefrontal cortex can be downloaded from http://resource.psychencode.org/. All software used in the study is publicly available as described in the Methods. The code for both single‐cell eQTL MR and colocalization analyses is now available on GitHub (https://github.com/gjw2025up/Single‐cell‐eQTL‐MR).
