# Supplementary material for: Integrating Genetic and Single‐Cell Genomic Data to Reveal Brain Cell‐Specific Regulation of Attention‐Deficit/Hyperactivity Disorder Risk in the Prefrontal Cortex
Source: Brain Behav. 2025 Jul 7;15(7):e70664. doi: 10.1002/brb3.70664 (PMC12230629; doi:10.1002/brb3.70664)

***Supplementary Figures***

Supplementary Figure 1 | **Spearman correlation estimates between MR effect sizes of genes on ADHD under different P value screening conditions (no threshold, P < 0.5, and P < 0.25).**

***
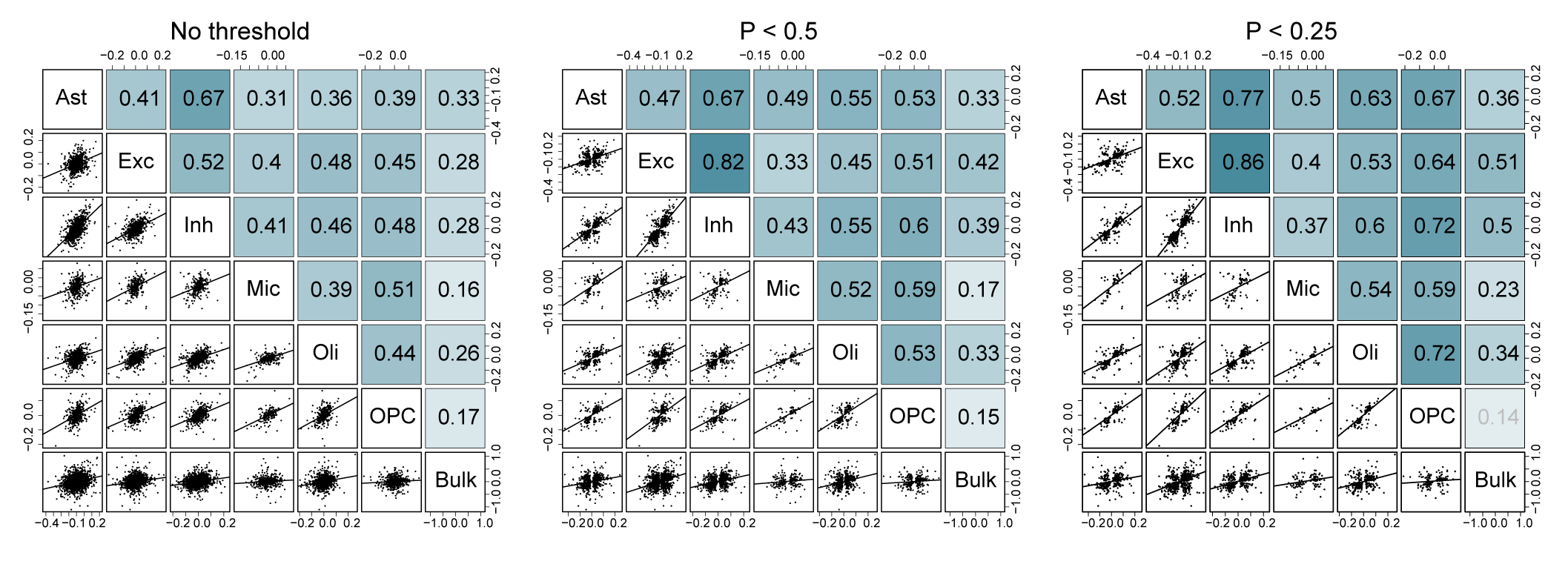
***

Supplementary Figure 2 | **The top 10 enriched terms of KEGG and GO enrichment analysis**. Colored bars correspond to terms with significant P values (<0.05).


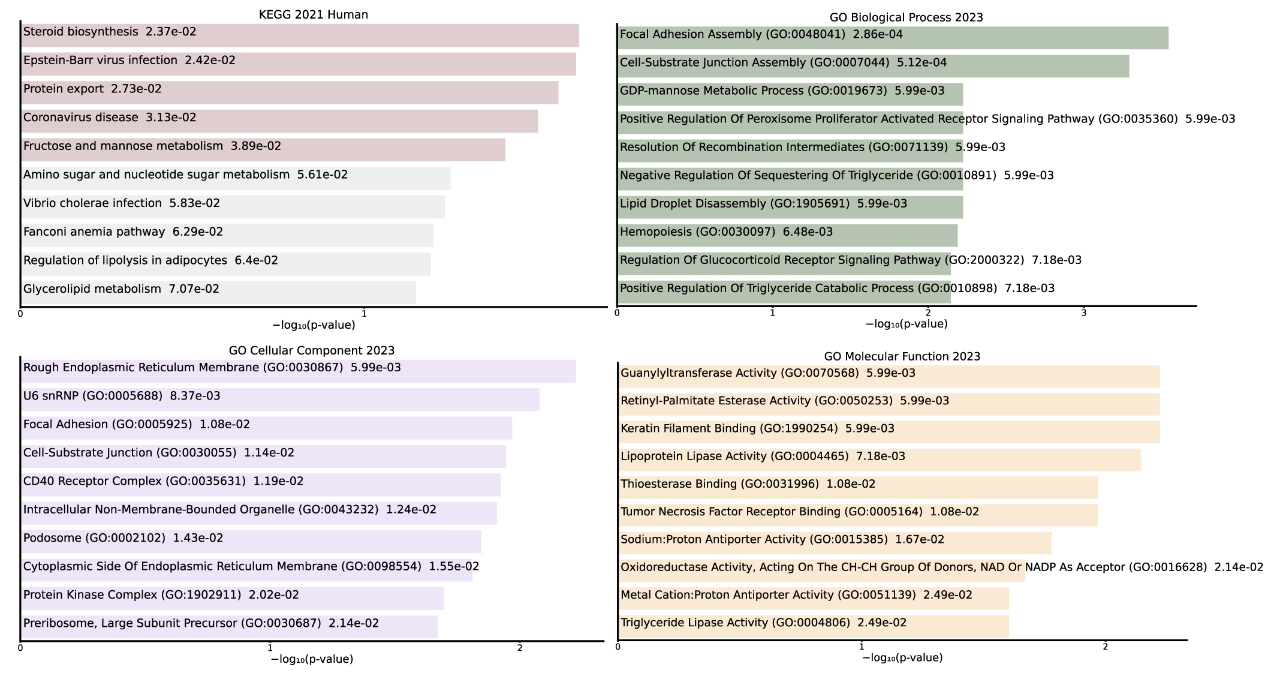


Supplementary Figure 3 | **The expression patterns of genes in the developing human cortex.**


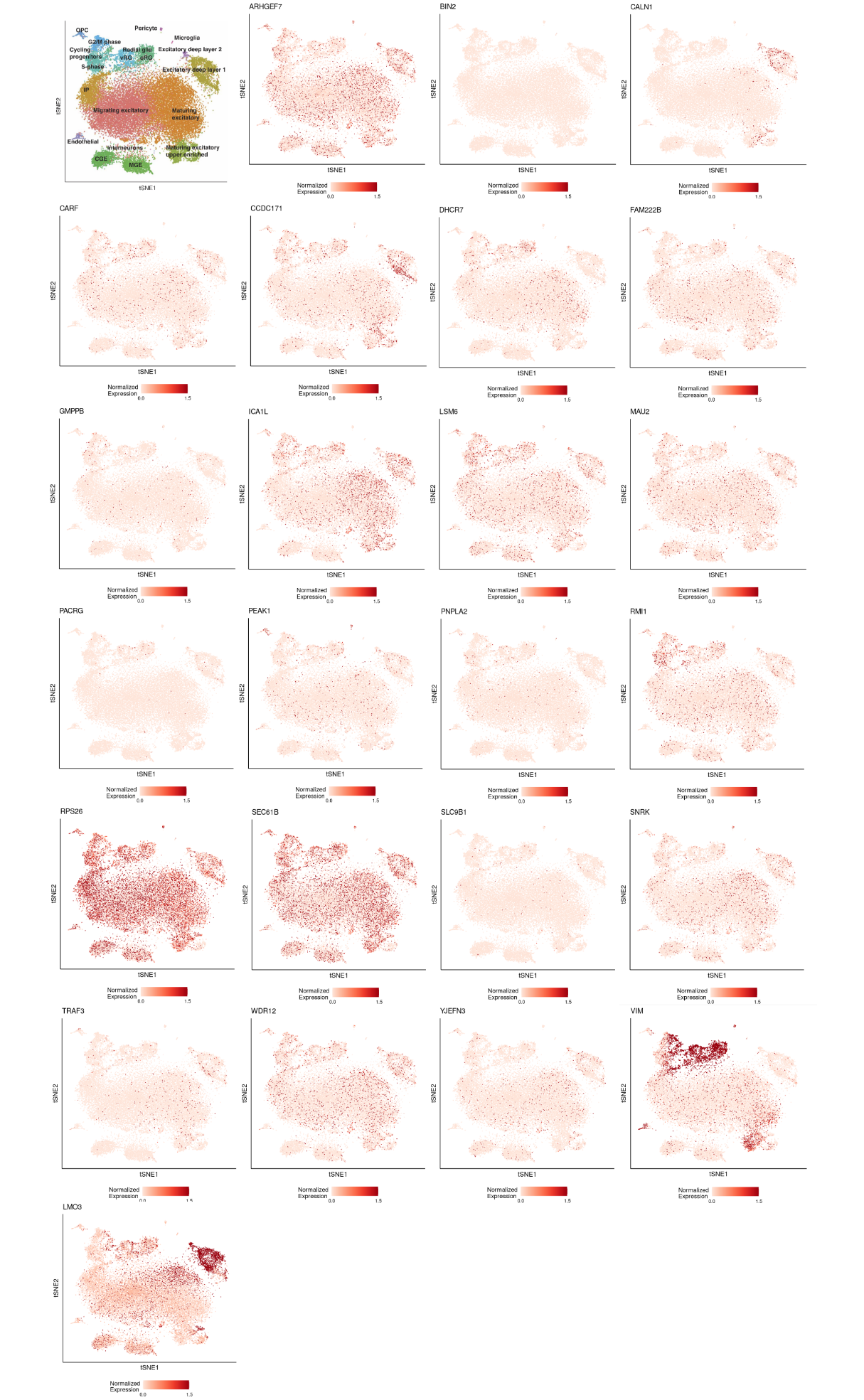

Supplement: Supplementary file 1 — Supplementary Figures: brb370664‐sup‐0001‐Figures.docx [file BRB3-15-e70664-s001.docx]
